# Feed Intake and Weight Changes in *Bos indicus-Bos taurus* Crossbred Steers Following Bovine Viral Diarrhea Virus Type 1b Challenge Under Production Conditions

**DOI:** 10.3390/pathogens6040066

**Published:** 2017-12-12

**Authors:** Chase A. Runyan, Erika D. Downey-Slinker, Julia F. Ridpath, Thomas B. Hairgrove, Jason E. Sawyer, Andy D. Herring

**Affiliations:** 1 Department of Animal Science, Texas A&M University, College Station, TX 77843, USA; chase.runyan@angelo.edu (C.A.R.); tbhairgrove@tamu.edu (T.B.H.); j-sawyer@tamu.edu (J.E.S.); 2 Department of Agriculture, Angelo State University, San Angelo, TX 76909-0888, USA; 3 Department of Veterinary Integrative Biosciences, Texas A&M University, College Station, TX 77843, USA; eddowney87@gmail.com; 4 Elanco Animal Health, Larchwood, IA 51241, USA; 5 USDA-ARS National Animal Disease Center, Ames, IA 50010, USA; ridpathconsulting@gmail.com; 6 Ridpath Consulting, LLC, Gilbert, IA 50105, USA; 7 Texas A&M AgriLife Extension, College Station, TX 77843, USA; 8 Texas A&M AgriLife Research, College Station, TX 77843, USA

**Keywords:** BVDV, Nellore-Angus, rectal temperature, vaccination, morbidity, feedlot performance

## Abstract

Bovine viral diarrhea virus (BVDV) has major impacts on beef cattle production worldwide, but the understanding of host animal genetic influence on illness is limited. This study evaluated rectal temperature, weight change and feed intake in *Bos indicus* crossbred steers (*n* = 366) that were challenged with BVDV Type 1b, and where family lines were stratified across three vaccine treatments of modified live (MLV), killed, (KV) or no vaccine (NON). Pyrexia classification based on 40.0 °C threshold following challenge and vaccine treatment were investigated for potential interactions with sire for weight change and feed intake following challenge. Pyrexia classification affected daily feed intake (ADFI, *p* = 0.05), and interacted with day (*p* < 0.001) for ADFI. Although low incidence of clinical signs was observed, there were marked reductions in average daily gain (ADG) and cumulative feed intake during the first 14 day post-challenge; ADG (CV of 104%) and feed efficiency were highly variable in the 14-day period immediately post-challenge as compared to the subsequent 14-day periods. A sire × vaccine strategy interaction affected ADFI (*p* < 0.001), and a sire by time period interaction affected ADG (*p* = 0.03) and total feed intake (*p* = 0.03). This study demonstrates that different coping responses may exist across genetic lines to the same pathogen, and that subclinical BVDV infection has a measurable impact on cattle production measures.

## 1. Introduction

Bovine respiratory disease (BRD) imposes a large financial burden on the U.S. cattle industry, with annual losses estimated to be from $750 million to over $1 billion [1,2,3], with newly-received, pathogen-naïve cattle at highest risk for illness. Visual signs plus rectal temperature over 40 °C (104.0° F) are considered indicative of BRD and are typical criteria for antimicrobial use [4]. Reductions in weight gain [5,6], profitability [7], and carcass value [8,9] have been associated with clinical BRD morbidity. Bovine viral diarrhea (BVD) refers to a range of clinical presentations, including respiratory disease, resulting from BVD virus (BVDV) infection [10,11]. Signs can be highly variable or subclinical depending on the virulence of the specific strain [12,13]. Regardless of clinical presentation, BVDV infection leads to suppression of various immune-associated defense mechanisms [12,14]. There are two species (BVDV1 and BVDV2), and within species there are different subgenotypes. BVDV1 subgenotype b (BVDV1b) is predominant in U.S. feedlots [15]. Investigations regarding cattle breed and family influences on BRD incidence [8,9,16,17] have shown selection potential for improved health, but are very limited, particularly for specific BRD pathogens. Recent findings have identified genomic regions affecting BRD incidence or responses [18,19,20,21], and improved understanding of complex host-pathogen relationships require detailed phenotypes and quantifiable causative components to advance potential cattle management strategies. The objective of this study was to investigate potential interactions of sire lines with BRD vaccine treatments on weight gain and feed intake following a standardized BVDV1b challenge under common U.S. cattle management and production conditions. We observed that febrile responses following challenge were minimal, yet weight gain, feed intake and feed efficiency (weight gain to feed intake ratio) were reduced, which correspond with previous lymphopenia, and thrombocytopenia results. We also observed several interactions involving vaccine treatment and/or sire for multiple performance measures that indicate complexity and diversity in animal response to a standardized BVDV challenge.

## 2. Materials and Methods

### 2.1. Animal Procedures

All animal procedures were reviewed and approved by the Texas A&M University Institutional Animal Care and Use Committee and the Texas A&M University Institutional Biosafety Committee. Yearling, half-blood (F_2_ and F_3_) Nelore-Angus steers from the Texas A&M University McGregor Genomics herd (a *Bos taurus-Bos indicus* crossbred population developed for genomic studies) were utilized. Steers for this project were spring-born, and were not vaccinated against bovine respiratory disease (BRD) pathogens as calves. Each year, animals were vaccinated for clostridial diseases, castrated while nursing dams, and were weaned at approximately 7 months’ age. Following weaning, calves were managed as single groups and remained on pasture or were fed a growing ration depending upon the year until being transported approximately 165 km from McGregor to College Station in January or February. Prior to vaccination, all steers were confirmed to be free of BVDV persistent infection by testing ear notch samples through immunohistochemistry or antigen-capture ELISA at the Texas Veterinary Medical Diagnostic Laboratory (TVMDL, Amarillo, TX, USA). Steers were confirmed seronegative to BVDV antibodies prior to vaccination by TVMDL through serum neutralization assay. Throughout this study low-stress cattle handling methods were emphasized during movement, processing, and data collection.

### 2.2. Vaccination and Challenge Protocols

Steers were stratified by sire and genomics cow family of the structured McGregor Genomics population [22] and assigned to one of three BRD vaccine strategies utilizing killed (KV), modified-live (MLV), or no (NON) vaccine at approximately 12 months’ age. Cattle were managed as a single contemporary group within each year. Table 1 shows distributions across vaccine groups and study year. Steers in the KV group received an initial dose of Vira Shield^®^ (Novartis Animal Health US, Inc., Greensboro, NC, USA) injection on d −56 or −49 pre-challenge and a booster on d −35, −28, or −25, depending on year, with a target of 21 d between priming and booster vaccinations. Steers in the MLV group were vaccinated with a single dose of Arsenal^®^ 4.1 (Novartis Animal Health US, Inc., Greensboro, NC, USA) on d −35, −28, or −25, depending on year, prior to the challenge (the same d of KV booster in each year). Both of these vaccines are licensed to aid in the control of infectious bovine rhinotracheitis, parainfluenza-3, bovine respiratory syncytial virus, and BVDV. The same vaccine products were used in all years according to label instructions, and the components of these products did not change during the study time frame. Steers in the NON group received neither BRD vaccination nor sham injection prior to BVDV challenge. Steers receiving MLV were kept separated from KV and NON-treated steers with no nose-to-nose contact possible for 7 to 10 d following treatment application. 

On d 0, all steers were challenged with a type 1b non-cytopathic BVDV strain (CA0401186a) obtained from the USDA-ARS National Animal Disease Center [23]. This strain was isolated from a PI calf and submitted to the NADC from the California Animal Health and Food Safety Laboratory in Tulare [24]. Each steer received 5 mL of inoculum (1 × 10^5^ TCID/mL). A 2.5 mL dose was placed in each nasal passage with the animal’s nose elevated until it was visually observed to have swallowed (indicating ingestion of pathogen solution). This particular strain of BVDV was chosen for this study because it had previously been reported to cause recognized immunological and febrile signs of morbidity, but to be of low risk for extreme illness or death [23]. Challenge dates were 11 May, 10 May, 15 May, and 4 June from 2010–2013, respectively.

Prior to challenge through 42 d post-challenge, the cattle were fed a high-fiber growing diet that consisted of approximately 31.5% corn, 36.5% chopped alfalfa hay, 24.5% dry distillers grains, 2.5% commercial premix, and 5% molasses (88% DM). Cattle were housed and fed at the Texas A&M University Beef Cattle Systems Research Unit in 4 pens equipped with a GrowSafe (GrowSafe Systems, Ltd., Airdrie, AB, Canada) feed intake and behavior monitoring system (4 bunk nodes per pen). Feed was delivered twice daily to ensure ad libitum access. Cattle were housed so that vaccine treatments and family lines were represented across the 4 feedlot pens with approximately 20 to 26 steers per pen, depending on the year. Cattle were acclimated to the diet for 6 to 10 weeks prior to the viral challenge d in each year; however, individual intake was not assessed until 7 to 10 d following MLV administration, at which time steers were placed into the pens in which they remained until 42 d post-challenge. Following the 42-d post-challenge evaluation period, cattle were transported to a commercial feedlot, fed approximately 160 to 180 d in a single pen each year, and subsequently harvested at a commercial beef processing plant. 

### 2.3. Sample and Data Collection

Body weights and rectal temperatures were recorded on d 0 (BVDV challenge), 3, 7, 10, 14, 28, and 42. Observed rectal temperature on d 3 to 14 was used for pyrexia classification (rectal temperature > 40.0 °C = pyrexia). Across collection d the order of processing pens was rotated. Weight gain and feed efficiency were evaluated according to three periods (d 0 to 14, d 14 to 28, and d 28 to 42) following challenge. Daily feed intake was recorded through the GrowSafe system software (GrowSafe Systems, Ltd., Airdrie, AB, Canada) recorded from midnight to midnight. 

Clinical observations were conducted twice daily for 14 d post-challenge to assess apparent health with a score of 0 (no signs), or 1 to 5 (least severe to most severe) for commonly associated signs of BRD/BVD (cough, ocular secretion, nasal secretion, depression, diarrhea, and gauntness/shrink). From d 15 to 42, observations were conducted once daily. The same personnel evaluated visual signs across all years. Animals exhibiting rectal temperatures over 40.0 °C were administered tulathromycin per label directions. 

### 2.4. Statistical Analyses

Mixed model procedures of SAS (SAS Inst. Inc., Cary, NC, USA), were utilized for all performance trait analyses through repeated measures that incorporated a first-order autoregressive covariance structure. Main effects (*p* < 0.15) and interaction terms (*p* > 0.25) found to be non-significant were removed for final analyses. Least squares means for responses of interest were compared following an indicative *F*-test (*p* ≤ 0.05) through two-tailed *t*-tests, but not all significant effects were compared. 

Models to investigate daily feed intake (DFI, as-fed basis) included fixed effects of day(nested within year), pen(nested within year), year, vaccine strategy, pyrexia status, two-factor interactions of day × vaccine strategy, day × pyrexia status, vaccine × pyrexia status, and the 3-way interaction of vaccine strategy × d × pyrexia status. 

Rectal temperature and weight (d 0, 3, 7, 10, 14, 28, 42) were analyzed with initial models that included potential effects of day (nested within year), pen (nested within year), year, vaccine strategy, and the two-way interaction of day × vaccine strategy. 

Average daily gain (ADG), cumulative feed intake (as-fed), and feed efficiency (weight gain to feed intake ratio, G:F, as-fed) were calculated for the 3 14-d periods following BVDV challenge as well as the 42-d period and were analyzed with models initially containing fixed effects of period nested within year, pen nested within year, vaccine strategy, pyrexia status, two-way interactions involving period with vaccine strategy and with pyrexia status, and period × vaccine strategy × pyrexia status. 

Frequency distributions of threshold rectal temperature status, and clinical symptom status across other study factors such as vaccine type, pen and year were evaluated through Chi-square analyses. 

To characterize potential sire group effects and interactions, a subset of the data was utilized where records were removed corresponding to sires with <2 progeny per vaccine strategy (35 records from 15 sires were removed). The same mixed models were utilized as described above, but with sire and sire × vaccine strategy investigated as additional fixed effects. Analyses of ADG, cumulative feed intake, and G:F ratio by period also investigated sire × period interaction. For all comparisons and conclusions, the statistical threshold was *p* < 0.05.

## 3. Results and Discussion

Distribution of pyrexia status (rectal temperature threshold ≤40.0 °C vs. >40.0 °C used for antimicrobial therapy) within 14 d post-challenge differed due to year (*p* = 0.003) and vaccine strategy (*p* < 0.001), but not pen (within year). Percentage of animals classified as being pyrexic across years ranged from a low of 45% in 2010 to a high of 72% in 2011; 47.2% of the MLV steers exceeded the threshold rectal temperature, lower than the rate observed in KV and NON steer groups (65.8% and 69.4%, respectively). Few clinical signs were observed; no cattle were identified with signs severe enough to merit removal from pens for additional evaluation or treatment. Only minor clinical signs (score of 1 or 2 on 0-to-5 point scale) of depression, coughing or gauntness were recorded, and these were on a very low percentage (14%, 51 of 364) of animals. Distributions of animals that showed none vs. minor visual signs of morbidity did not differ across year, pen, or rectal temperature threshold. Among the 51 steers that exhibited any clinical sign, vaccine groups tended to differ (*p* = 0.07) where 47.1% (*n* = 24) were non-vaccinated steers compared to 25.5% in the KV and 27.5% in the MLV groups. Downey-Slinker et al. [25] described antibody titer, lymphopenia, and thrombocytopenia in these cattle and found lymphopenia and thrombocytopenia to be much more pronounced in NON vs. vaccinated groups even at similar antibody and clinical sign status.

Daily feed intake was analyzed with similar models, and d nested within year, pen nested within year, and year accounted for variation (*p* < 0.001). Vaccine strategy affected daily feed intake (*p* < 0.01) as did pyrexia classification (*p* = 0.05). The interaction of pyrexia status with d was important for ADFI (*p* < 0.001) as well. The interaction of pyrexia status with vaccine strategy and the interaction of vaccine strategy with d were not important for ADFI (*p* > 0.10). The three-way interaction of d × vaccine strategy × pyrexia status was not significant (*p* > 0.10) for ADFI. 

**Figure 1:**
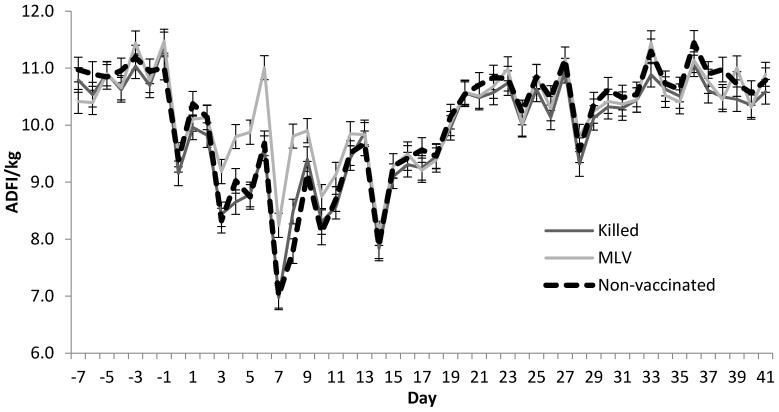
Vaccine strategy × d interaction least squares means for daily feed intake (above, *p* < 0.001). Animals were removed from their pen for approximately 1 h to obtain weight, rectal temperature and blood samples on d 0, 3, 7, 10, 14, 28 and 42. Least significant difference values (*p* < 0.05) for comparisons across d are approximately 0.5 kg.

Figure 1 displays least squares means for the vaccine strategy × d interactions in daily feed intake. For ADFI no differences existed for d 0–2; however, MLV steers ate 0.8 to 1.3 kg more per d (*p* < 0.05) than KV and NON steers for d 3 through 8. On d 9 and 10, steers in the MLV group ate more than steers in the NON group with KV intermediate. No differences in ADFI were observed among the vaccine groups for d 11–14.

**Figure 2:**
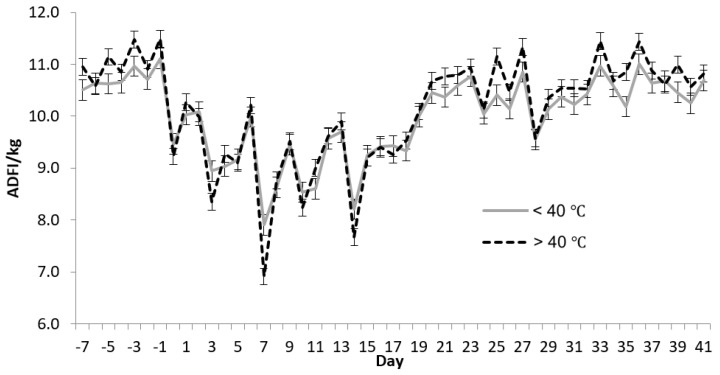
Daily feed intake (*p* < 0.001) least squares means for d × pyrexia status combinations Animals were removed from their pen for approximately 1 h to obtain weight, rectal temperature and blood samples on d 0, 3, 7, 10, 14, 28 and 42. Least significant difference values (*p* < 0.05) for comparisons across d are approximately 0.5 kg for daily feed intake.

Weight and rectal temperature were analyzed as repeated measures with similar models for the seven scheduled evaluation times (d 0, 3, 7, 10, 14, 28, 42). Only d within year (*p* < 0.001) and year (*p* < 0.001) explained variation in body weight (no vaccine strategy or interaction with vaccine strategy effects). Rectal temperature differences were observed due to vaccine strategy (*p* < 0.01), pen within year (*p* < 0.01) and vaccine strategy × d combination (*p* < 0.001, Table 2) in addition to d within year (*p* < 0.001) and year (*p* < 0.001). The magnitude of differences in rectal temperature was quite small despite being statistically significant. Figure 2 shows the ADFI patterns across the two pyrexia classifications. 

The distribution of rectal temperatures did not correspond to clinical observations. It is speculated that in these cattle, rectal temperatures may have been more indicative of their temperament [22] and/or stress responsiveness rather than outright illness, particularly because several animals exhibited elevated temperature on d 0 with no visual signs of illness, showed no measurable titers to BVDV or IBR (data not shown), and differences in rectal temperature between year seemed to correspond with observed cattle behaviors associated with routine handling.

Average daily gain, cumulative feed consumption and G:F were analyzed with similar models as repeated measures across 3 14-d time periods following BVDV challenge. Period within year, pen within year, and year effects were influential for all three of these traits (*p* < 0.01). Neither pyrexia status nor its interaction with time period affected these responses (*p* > 0.10). 

**Figure 3:**
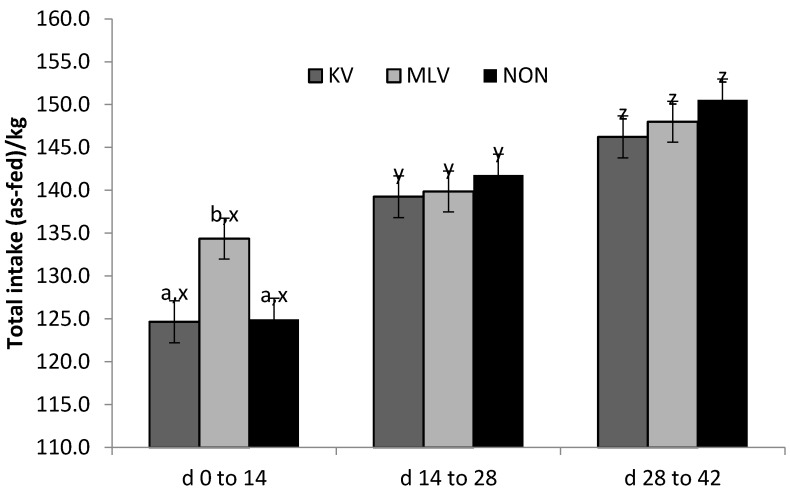
Least squares means for vaccine × time period interaction (*p* < 0.001) on total feed intake following BVDV challenge at d 0. KV = killed, MLV = modified live, and NON = non-vaccinated. Letters a-c and x-z designate differences (*p* < 0.05) across vaccines within time period and across time periods within vaccine, respectively.

**Figure 4:**
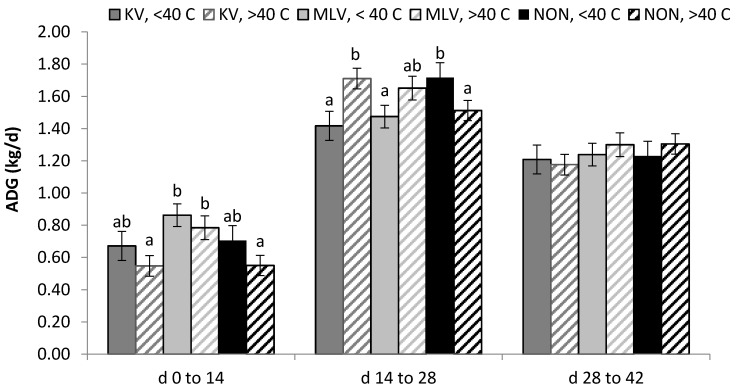
Least squares means for the vaccine × pyrexia status × time period interaction (*p* = 0.049) on ADG following BVDV challenge at d 0. KV = killed, MLV = modified live, and NON = non-vaccinated. Letters a-c designate differences (*p* < 0.05) for vaccine and pyrexia classification within time period. All vaccine-pyrexia combinations differed (*p* < 0.05) across all 3 time periods except for KV < 40 °C between second and third periods. LSD (*p* < 0.05) was approximately 0.23 kg.

Vaccine strategy × period interaction influenced total feed intake (*p* < 0.001, Figure 3) but not ADG or G:F; this interaction was driven by MLV steers having higher (*p* < 0.05) feed intake in the first 14 d following challenge than KV and NON-vaccinated steers. No intake differences existed among vaccine strategies during the second or third 14-d periods. The 3-way interaction of vaccine × period × rectal temperature threshold status affected ADG (*p* = 0.05, Figure 4), but not cumulative intake or G:F. The effect on ADG resulted from re-ranking of vaccine-pyrexia combinations in the first vs. the second 14-d period following challenge; NON-vaccinated steers experiencing pyrexia were consistently the lowest ranked group for ADG. No differences among vaccine-pyrexia combinations existed in the third period (d 28 to 42). 

**Figure 5:**
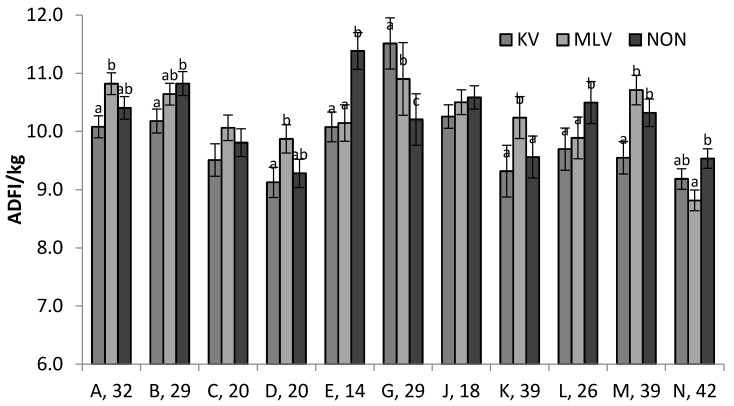
Sire × vaccine strategy interactions for ADFI (*p* < 0.001). KV = killed, MLV = modified live, and NON = non-vaccinated. Letters A-N denote sires followed by number of progeny (only sires with ≥4 progeny per vaccine strategy compared). Letters a–c denote differences (*p* < 0.05) among vaccines within sire group.

A notable pattern is evident in Figure 4 and Figure 5 and in Table 3 regarding cumulative intake, ADG and G:F during the first 14 d following BVDV challenge. Differences in intake were pronounced across time periods and steadily increased from period 1 to period 3. However, the overall increases in ADG and G:F were greater between periods 1 and 2 than between period 2 and 3, and likely reflect compensatory gain. 

Most trials evaluating BRD effects on feedlot performance have not identified specific disease pathogens and have targeted newly received cattle, but there are some similar observations from the present trial to other reports regarding weight gain and feeding responses. We observed depressed intake during first 14 d after BVDV challenge. Hutcheson and Cole [26] summarized 18 experiments where overall 94.6% of “healthy” calves were observed eating as compared to 83.4% of calves classified as “morbid,” and the “morbid” vs. “healthy” calves had lower feed intake (0.9% vs. 1.65% BW) in the first 7 d after arrival. 

We observed reduced ADG in the first 14-d period following BVDV challenge vs. later time periods, and, consistently lower ranking ADG in cattle exhibiting pyrexia in the first 14-d period; differences within vaccine group were not statistically different. Schneider et al. [9] found BRD to decrease feedlot ADG after arrival by 0.28 kg/d. Reduction in feedlot ADG due to BRD incidence has been reported in several instances for the entire finishing period: −0.06 kg/d in treated vs. untreated based on visual signs and rectal temperature greater than 40°C [5], −0.25 kg/d for treated vs. non-treated based on visual diagnosis [6], and −0.07 kg/d for treated cattle or presence of lung lesions at harvest vs. non-treated animals with no lung lesions [9]. 

When assessing BRD morbidity status and its impacts on performance, the different criteria utilized for morbidity classification must be considered. The three studies cited in the preceding paragraph as well as others [27,28,29] have reported very different proportions of cattle with lung lesions at harvest vs. those diagnosed/treated for BRD during the feeding period, indicating that measures of clinical morbidity may not be accurate indicators of BRD status or impact. Literature reports are much more limited regarding feedlot performance pertaining specifically to BVDV comparisons (as opposed to the more general designation of “BRD”). Schunicht et al. [30] observed increased ADG, and daily feed intake in cattle administered BVDV-containing vaccine vs. those receiving no BVDV vaccine. Booker et al. [31] studied BVDV infection on calves at feedlot arrival and found varying rates for BRD signs with or without fever due to type of BVDV infection. Rectal temperature on evaluation d in the present study seems to be imprecisely associated with animal health.

As potential genetic influences were of interest and sire stratification was incorporated into the experimental design, a subset of data was evaluated. Observations from sires with <2 progeny per vaccine strategy were removed (total of 35 animals in the study); sire and sire interaction terms were added to the initial models. Sire and sire × vaccine strategy affected daily feed intake (*p* < 0.001) and weight (*p* < 0.001). Sire resulted in differences in rectal temperature (*p* < 0.001), but sire × vaccine strategy did not (*p* > 0.10). Only 2 sire groups had no differences in ADFI due to vaccine allocation. Some sire groups had 0.6 to 1.2 kg/d. (*p* < 0.05) difference in ADFI across vaccine strategies, and 5 sires had higher ADFI in non-vaccinated progeny as compared to at least one of the vaccinated groups (Figure 5). 

**Figure 6:**
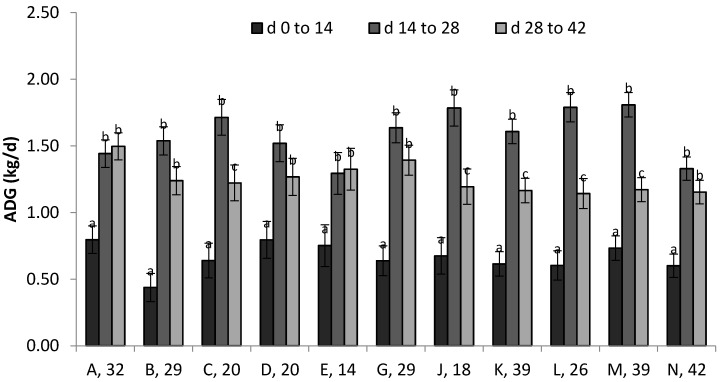

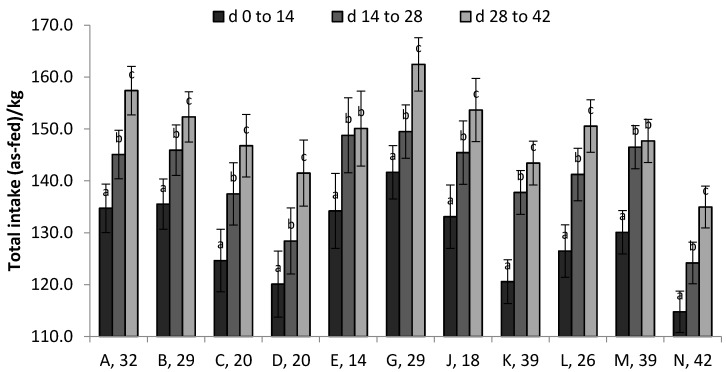
Sire × time period interactions for ADG (*p* = 0.030) and total feed intake (*p* = 0.032) following BVDV challenge. Letters A–N denote sires, followed by number of progeny (only sires with ≥4 progeny per vaccine strategy compared). Letters a-c designate differences (*p* < 0.05) across time periods within sire groups.

As these traits measured daily encompassed the entire 49 d period (from d −7 to 41), there was potential for compensating or recovery from initial effects associated with the BVDV challenge. As a result, performance traits pooled by 14-d period may be more illustrative than daily observations over the entire study. Sire tended to influence ADG (*p* = 0.07) and affected cumulative feed intake (*p* < 0.01), but did not influence G:F in the 14-d periods. The sire × vaccine strategy combination did not affect these three traits; however, a sire × time period interaction existed for ADG (*p* = 0.03) and total feed intake (*p* = 0.03), displayed in Figure 6. The same general trend can be seen across sire groups where ADG was much lower in the first 14-d period, increased substantially during the second 14-d period and was reduced in the third period. The magnitude of difference among periods varied across sires, resulting in the observed interaction. Similarly, the same trend for intake to increase steadily across all three periods can also be seen across the sire groups but with magnitude of difference varying by sire, resulting in the observed interaction. These types of comparisons among genetic lines are of interest as they may provide ways to identify animals that have different physiological responses to pathogen exposure. 

Genetic investigations of BRD incidence have produced low heritability estimates [9,16,32]. Some reasons for low estimates are likely at least in part to inaccuracy of using visual signs [33], which can be influenced from prey animals not exhibiting signs of weakness [34], particularly when unaccustomed to settings or personnel. However, it is also plausible that some animals could be responding differently physiologically to the same pathogen, and/or, across time and space different pathogen combinations. Schneider et al. [9] reported heritability estimates of BRD incidence and the number of treatments (during the feedlot phase) of 0.07 and 0.02, respectively. Snowder et al. [32] postulated that disease incidence variability in most field settings cannot be explicitly attributed to differences in animal response versus differences in pathogenic exposure. It is not unreasonable to postulate that compensating biological mechanisms that may be different across animals and can contribute to low heritability estimates for these types of complex traits. Neibergs et al. [35] estimated heritability of BRD incidence to be 17% and 29% when using case-control or graduated scoring analyses, respectively, as opposed to binomial (yes or no) morbidity classification. Recent findings of QTL associated with BVD persistent infection [18] and BRD susceptibility or response [19,20,21] also provide evidence for continued genetic investigation regarding host-pathogen interactions. Increases in U.S. feedlot mortality from the early 1990s into the 2000s [36,37] attributed to BRD is perplexing given improved vaccination recommendations and implementation of beef quality assurance programs. Larson [38] pondered whether or not cattle health may have been compromised through intense selection for growth and stated that large datasets with parentage and health information were needed to study genetic impacts of health. However, the complexity of these kinds of phenotypes, and the undocumented subclinical attributes of these diseases also likely contribute to either low heritability estimates or un-improved morbidity rates. Keele et al. [39] stated the formidable complexity in describing genomic associations with cattle lung lesions at harvest. In addition to genetic influences and vaccination strategies, and traditional diagnostic strategies, eating and drinking behaviors have also been associated with BRD [40,41,42]. For improved understanding of BVDV, large increases in diagnostic and/or descriptive efficiency are likely to come from whole system approaches to collectively evaluate combinations of host genetics, health management, and animal behavioral descriptors, as opposed to any of these solely.

## 4. Conclusions

Overall, vaccination protocols improved measures of performance even in the absence of substantial clinical illness, suggesting that some protection was conferred to pathogen exposure, and corresponded to lymphopenia, and thrombocytopenia results [25]. The interactions observed between pyrexia status and vaccination protocol also suggest that rectal temperature, particularly in *Bos indicus* influenced cattle may not be a functional indicator of ‘well-being’, as vaccinated animals with pyrexia displayed some improvements in measures of performance. This may be a manifestation of greater immunostimulation and therefore greater resilience to the challenge, with the febrile response actually indicating a successful outcome rather than a negative outcome. Although sire differences were documented, the observed interactions suggest that the genetic components of resilience to a standardized BVDV1b challenge may not only be complex, but diverse across families. Evaluation of suites of immunological and animal performance responses to BVDV infection are needed to better describe physiological axes and determine optimized genetic × management combinations in beef cattle production systems.

## Figures and Tables

**Table 1:** Distribution of animals across bovine respiratory disease vaccine strategy and study year.

Vaccine Strategy ^1^	Year				
	2010	2011	2012	2013	Totals
Killed	28	34	30	28	120
Modified live	25	35	33	32	125
Non-vaccinated	25	35	33	28	121
Totals	78	104	96	88	366

^1^ The stratification of vaccine strategy across sire groups within each year resulted in less than perfect balance across overall vaccine treatments.

**Table 2:** Rectal temperature (°C) least squares means ^1^ for vaccine strategy and study day combinations.

Vaccine	Trial Day						
	0	3	7	10	14	28	42
Killed	39.8	39.9	39.9	39.6	39.7	39.7	39.8
Modified live	39.8	39.6	39.7	39.6	39.6	39.8	39.8
Non-vaccinated	39.8	40.1	39.9	39.6	39.7	39.8	39.8

^1^ SEM of 0.04 to 0.05. Pyrexia classification used for analyses was based on rectal temperature threshold of ≤40.0 °C or >40.0 °C at any d (3, 7, 10, or 14) post-BVDV challenge; no distinction was made for steers with elevated temperature on d 0 prior to challenge, or for steers with single vs. multiple d during d 3 to 14, or, on d 28 or 42.

**Table 3:** Summary statistics of variables evaluated during 42-d following BVDV challenge.

Variable ^1^	Mean	SD	CV	Minimum	Maximum
Weight, kg	343.2	51.03	14.9	188.7	526.1
ADG d 0 to 14, kg	0.67	0.692	103.7	−1.88	3.04
ADG d 14 to 28, kg	1.57	0.603	38.4	−1.62	3.24
ADG d 28 to 42, kg	1.25	0.491	39.2	−0.65	3.04
ADG d 0 to 42, kg	1.17	0.326	28.0	−0.43	1.90
Daily feed intake, kg	10.1	2.70	26.7	0.0	21.0
Intake d 0 to 14, kg	127.8	28.10	22.0	30.3	215.0
Intake d 14 to 28, kg	140.5	28.46	20.3	23.0	230.9
Intake d 28 to 42, kg	149.3	28.48	19.1	71.3	243.7
Intake d 0 to 42, kg	418.0	79.59	19.0	154.9	689.6
G:F d 0 to 14	0.070	0.0826	118.2	−0.321	0.377
G:F d 14 to 28	0.157	0.0880	56.0	−0.986	0.495
G:F d 28 to 42	0.119	0.0498	41.7	−0.121	0.359
G:F d 0 to 42	0.118	0.0351	29.7	−0.071	0.296
Rectal temperature (°C)	39.8	0.52	1.3	37.8	42.3

^1^ Weight, rectal temperature evaluated on d 0, 3, 7, 10, 14, 28 and 42; individual feed intake recorded daily for d −7 to 42; ADG = average daily weight gain, G:F = weight gain to feed intake ratio. All animals received BVDV1b challenge on d 0.
